# Theoretical Considerations and the Microelectrophoresis Experiment on the Influence of Selected Chaotropic Anions on Phosphatidylcholine Membrane Surface Charge Density

**DOI:** 10.3390/molecules25010132

**Published:** 2019-12-29

**Authors:** Joanna Kotyńska, Monika Naumowicz

**Affiliations:** Laboratory of Bioelectrochemistry, Department of Physical Chemistry, Faculty of Chemistry, University of Bialystok, K. Ciolkowskiego 1K, 15-245 Bialystok, Poland; monikan@uwb.edu.pl

**Keywords:** chaotropic anions, liposomes, phosphatidylcholine, surface charge density, binding constants

## Abstract

Influence of sodium salts of selected chaotropic anions from the Hofmeister series (NaCl, NaBr, NaNO_3_, NaI) on the surface charge density of phosphatidylcholine membranes was studied. Small unilamellar lipid vesicles were used as a model system in the investigations. The theoretical and experimental approach to the interactions between inorganic anions and phosphatidylcholine membranes is presented. Experimental membrane surface charge densities data were determined as a function of pH of the aqueous electrolytes using microelectrophoresis method. The quantitative description of the interactions between zwitterionic phosphatidylcholine membrane and monovalent anions is presented. The equilibria constants of the binding of solution ions onto phospholipid surface were calculated. Knowledge of these parameters was essential to determine the theoretical membrane surface charge density values. The theoretical data were compared to the experimental ones in order to verify the mathematical model. Both approaches indicate that the anion-phosphatidylcholine membrane interaction increases with the size of the anion. The adsorption of chaotropic anions to membranes was found to follow the Hofmeister series I^−^ > NO_3_^−^ > Br^−^ > Cl^−^.

## 1. Introduction

Lipids are essential biomolecules in the function and structure of living matter. These molecules have amphiphilic properties and are building blocks of cells as well as model membranes. Since natural membranes represent complicated assemblies of molecules, their study is extremely involved. To facilitate this task, the properties of natural membranes are often studied using liposomes. The most widely used lipid in liposome work is phosphatidylcholine (PC), a zwitterionic phospholipid, which at physiological values of pH, possesses one positive and one negative charge [1].

Interactions of biological membranes with electrolyte solutions modify the functioning of many membrane-related physiological processes, affecting the structure, dynamics, and stability of membranes. In the binding of electrolyte ions to membranes, ionogenic groups of membrane lipids are directly involved. Despite the widespread view that zwitterionic phospholipids do not interact strongly with the ions (especially compared to charged ones, e.g., phosphatidylserine), the phenomenon has been intensively studied. Numerous experimental studies, including those on ^1^H-NMR [2], ^2^H-NMR, Raman spectroscopy [3], Langmuir method [4], electrochemical impedance spectroscopy [5], chronopotentiometry [6], cyclic voltammetry and chronoamperometry [7], microelectrophoresis [8,9,10], were made. In addition, simulation studies, including those on molecular dynamics simulations [11,12,13], were performed. However a variety of literature studies concentrate on cations, due to the importance and biological functions of mono- (Na^+^, K^+^) and divalent (Ca^2+^, Mg^2+^) ions [14,15,16]. In contrast, fewer studies have focused on effects of anions on the properties of lipid membranes and the obtained results are presented in a rather limited way [17,18,19,20,21]. Investigations have shown that for the common halide anions the adsorption to phosphatidylcholine membranes follows the Hofmeister series. Clarke and Lüpfert showed that anions adsorb on PC liposomes and reduce the membrane dipole potential with an order of effectiveness that corresponds to the Hofmeister series [20]. Vacha et al. observed stronger affinity of anions for the membrane from Cl^−^, to Br^−^ and to I^−^, so anions with large size penetrate deeper into the membrane than smaller ones. The authors have found that binding of anions with phospholipids is affected by the types of counterions. For example, the adsorption of Cl^−^ to bilayers is stronger when K^+^ is replaced by Na^+^ as the counterion [13]. Also, Jendrasiak, using the ^1^H-NMR method, showed that the strength of association of anions to zwitterionic lipids (egg phosphatidylcholine, dioleoylphosphatidylcholine, and lyso-egg phosphatidylcholine) follows the Hofmeister series [2]. Assuming that solution ions are adsorbed on the membrane surfaces, it is possible to determinate the association constants of the ions to the lipids. One of the methods to study ion-membrane interactions, which is of particular importance, is microelectrophoresis. Electrophoretic mobility measurements are widely used to characterize the surface charge of the membrane, a parameter which plays a critical role in processes, such as active transport, signaling, selective permeability. Since adsorption of ions on liposome surfaces affects the electrophoretic mobility of liposomes; the method is also used in quantitative descriptions of the binding phenomenon of ions to natural as well as model membranes [16,17,18,19,20,21,22]. Tatulian applied microelectrophoresis to the study of the association between alkaline metal cations and several anions with phosphatidylcholine liposomes. Values of surface densities of binding sites and binding constants of ions to liposome membranes were determined [8]. Interactions of metal cations with both one-and two component liposomal membrane surfaces have been also investigated with microelectrophoresis by our group (Figaszewski and co-workers) [9,22,23,24]. A different theoretical models quantitatively describing adsorption of solution ions on liposomal surfaces were presented and association constants between the ions and lipids were determined.

The major aim of the present paper is to investigate the effect of a range of anions on the electric properties of egg phosphatidylcholine liposomal membranes, using the microelectrophoresis method, for the determination of surface charge density. The dependence of the membrane surface charge density on pH (pH range of 2 to 9.5) of the electrolyte solutions was determined. 155 mM sodium salt solutions (NaCl, NaBr, NaI, and NaNO_3_) were used as electrolyte solutions. The quantitative characteristics of the equilibria between zwitterionic phosphatidylcholine membrane and selected anions was presented. The four-equilibrium mathematical model, proposed previously by our group and published [22], was used to describe the equilibria. Using this model, the binding constants of ions to ionogenic groups of phosphatidylcholine were calculated. Knowledge of these parameters was necessary to calculate theoretical membrane surface charge density values. Then, the four-equilibria model was validated by comparing the theoretical and experimental data.

## 2. Theory

In an ionizing liquid, a colloidal particle is surrounded by an ionic atmosphere composed chiefly of oppositely charged single ions, and that in considering the motion of the large particle it is necessary also to consider the effect of the atmosphere. In certain extreme cases (e.g., large non-conducting particles), the ionic atmosphere may be simply treated. The electrophoretic behaviour of the particle is strongly influenced by the size of the electrical double layer. If the thickness of the diffuse double layer is much smaller than the radius of curvature at any point on the surface (i.e., d << a), it is possible to consider the particle with its double layer as a parallel plate condenser whose plates are at distance apart given by the thickness (d) of the diffuse double layer. Let the plates have a charge q per unit area. When a steady state is reached in which particle is moving at a constant speed through the liquid, there is equal between frictional and electrical forces [25].

From the definitions of viscosity, velocity and mobility we obtain:

(1) $$\delta =\;\frac{\eta \;\mu }{d}$$

Making use of the electrostatic expression:

(2) $$\delta =\;\frac{\varepsilon \;\varepsilon_{0}}{4\;\pi \;d}.\;$$

Furthermore, introducing the mobility, we obtain the Smoluchowski equation:

(3) $$\mu =\;\frac{\varepsilon \;\varepsilon_{0}\zeta }{4\;\pi \;\eta }$$

where: *µ* is the electrophoretic mobility, *ε* is the relative permittivity of electrolyte, *ε*_0_ is the permittivity of free space, *ζ* is the zeta potential, *η* is the viscosity of the medium, *d* is the thickness of diffuse double layer.

It follows from the above expression that the electrophoretic mobility of non- conducting particle for which the ratio of particle radius to double layer thickness is large at all points on the surface is independent of its shape and size. The membranes surface charge densities from electrophoretic mobility measurements were calculated according to Equation (1) which is conversion of Smoluchowski equation for large non-conducting particles. Liposomes are large relative to the double layer thickness even at relatively low ionic strength so that the equation is accepted to describe their electrophoretic behaviour. We performed electrophoretic measurements in aqueous media and moderate electrolyte concentration (155 mM) that is why f(Ka) in this case is 1.5 and is referred to as the Smoluchowski approximation. Our systems fit the Smoluchowski model, i.e., particles are large (about 0.2 microns) dispersed in electrolytes containing more than 10^−3^ molar salt.

The quantitative description of the interactions between zwitterionic phospholipid membrane and solution monovalent ions was proposed by our group [22]. The four-equilibrium model was used to describe the dependence of the surface charge density of phosphatidylcholine liposomal membrane on the pH of the sodium chloride solution—the mathematical formulation of this model is presented in full details in the above-mentioned work. In the considered phosphatidylcholine-sodium salts (NaCl, NaBr, NaNO_3_, NaI) system, the following interactions has been assumed; two equilibria associated with the negative group (–PO^(−)^) of PC with H^+^ and Na^+^ ions. The other equilibria are associated with the positive group (–N^(+)^(CH_3_)_3_) of PC, with OH^−^ and X^−^ (X^−^ = Cl^−^, Br^−^, NO_3_^−^, I^−^) ions.

Assumptions of the model are described by Equations (4)–(7).

(4) $$A^{-}+H^{+}\Leftrightarrow \mathrm{AH}$$

(5) $$A^{-}+{\mathrm{Na}}^{+}\Leftrightarrow \mathrm{ANa}$$

(6) $$B^{+}+{\mathrm{OH}}^{-}\Leftrightarrow \mathrm{BOH}$$

(7) $$B^{+}+X^{-}\Leftrightarrow \mathrm{BX}$$

The association constant for H^+^ with –PO^(−)^ group: K_AH_ is expressed by the equation:

(8) $$K_{AH}=\frac{a_{AH}}{a_{A-\cdot \;}a_{H+}}$$

Equations for the association constants: K_ANa_, K_BOH_, K_BX_ are analogous to Equation (8).

(9) $$a_{A-}+\;a_{AH}\;+\;a_{ANa}\;=\;C_{PC}$$

(10) $$a_{B+}\;+\;a_{BOH}+\;a_{BX}\;=\;C_{PC}$$

(11) $$\delta \;=\;(a_{B+}\;-\;a_{A-})\;F$$

Assuming that the surface area occupied by a single PC molecule is 70 Å^2^ per molecule [26], the surface concentration of PC was determined [22].

Final equations:

- Equations describing surface charge density of phosphatidylcholine liposomal membrane:

(12) $$\frac{\delta }{F}=\;\frac{C_{PC}}{1+K_{BOH}a_{OH-}+\;K_{BX}a_{X-}}-\frac{C_{PC}}{1+K_{AH}a_{H+}+\;K_{ANa}a_{Na+}}$$

- Linear equations obtained by simplification of Equation (12) valid for high (Equation (13)) and low (Equation (14)) concentration of hydrogen ions:

(13) $$\frac{\delta \cdot a_{H+}}{F}=\;-\left[\frac{C_{PC}}{1+K_{BX}a_{X-}}\right]\cdot a_{H+}-\;\left[\frac{C_{PC}K_{BOH}K_{W}}{\left(1+K_{BX}a_{X-}\right){\;}^{2}{}_{}}+\frac{C_{PC}}{K_{AH}}\right]$$

(14) $$\frac{\delta }{F\cdot a_{H+}}=\;-\left[\frac{C_{PC}}{1+K_{ANa}a_{Na+}}\right]\cdot \frac{1}{a_{H+}}+\;\left[\frac{C_{PC}}{K_{BOH}K_{W}}+\frac{C_{PC}K_{AH}}{{\left(1+K_{ANa}a_{Na+}\right)}^{2}}\right]$$

where: A^−^ is group –PO^(−)^, B^+^ is group –N^(+)^(CH_3_)_3_, of phosphatidylcholine, $a_{A-}$, $a_{AH}$, $a_{ANa}$. , $a_{B+}$, $a_{BOH}$, $a_{BX}$—surface concentrations of particular groups on the membrane surface [mol m^−2^], $a_{H+}$, $a_{Na+}$, $a_{OH-}$, $a_{X-}$—volumetric concentrations of the solution ions [mol m^−3^], C_PC_—surface concentration of phosphatidylcholine.

Based on Equations (13) and (14) the coefficients describing these linear functions were determined and then subsequently used to calculate binding constants. Then, Equation (12) was used to determine theoretical liposome membrane surface charge values which were compared to experimental ones to verify the four-equilibrium model.

## 3. Results and Discussion

**Figure 1:**
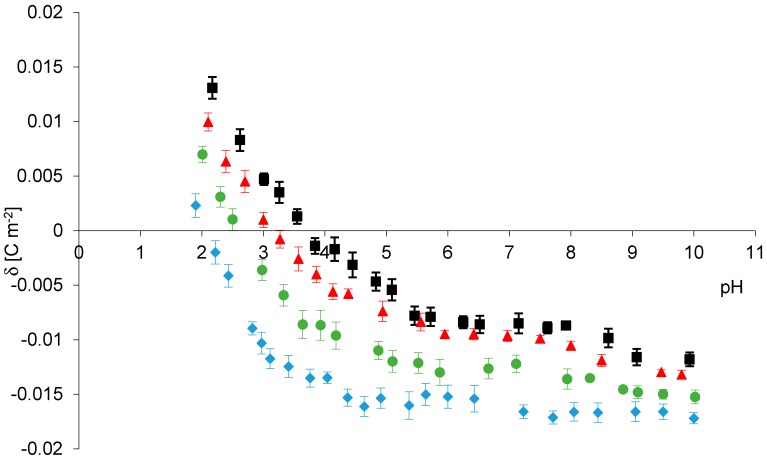
Experimental surface charge densities for phosphatidylcholine liposomal membranes as a function of electrolyte pH (155 mM); NaCl (black), NaBr (red), NaNO_3_ (green), NaI (blue).

Experimental results for phosphatidylcholine liposomes in four different salts solutions NaCl, NaBr, NaNO_3_ NaI are compared to each other in Figure 1. Depicted in the Figure are the curves of the dependence of PC membranes surface charge densities on pH of the electrolyte solutions. The data with NaCl as an electrolyte were obtained by our group previously [22]. As additional data, the size, polydispersity index (PDI) and zeta potential values of phosphatidylcholine liposomes dispersed in four different ionic solutions of polarizable anions are shown in Table 1.

As can be seen from the table, liposomes prepared in different electrolyte solutions are characterized by similar size distribution, irrespective of the type of the sodium salt. Liposomes exhibit a bimodal size distribution profile, with one population (representing approximately 5%–15% of all particles) with size between 30–50 nm and the other (representing about 85%–95% of the particles) with size between 160–215 nm. PDI measures the extent of homogenity/heterogenity in size distribution. PDI values indicate that phosphatidylcholine liposomes dispersed in studied sodium salt solutions are of a polydisperse nature. It should be noted that size distribution of liposomes determined at the time of their preparation can change upon their storage. Independently of the preparation method, liposomes tend to fuse and grow into bigger vesicles, which is a thermodynamically more favorable state. We repeated size measurements after an hour and size distributions were repeatable, therefore it may be concluded that PC liposomes dispersed in sodium salt solutions are relatively stable.

As shown in Figure 1, the surface charge density curves have a similar course for all sodium salts. However there are noticeable differences between the surface charge densities values of the membranes (including standard deviations) in whole pH range. At low pH values, the highest positive surface charge were obtained for NaCl, whereas the lowest were for NaI. At high pH values, the highest negative surface charge were obtained when using NaI as the electrolyte, while the lowest is NaCl. The results give evidence that analyzed anions also significantly influence on shift in isoelectric point of phosphatidylcholine membrane, from pH ~2.1 for NaI to pH ~3.8 for NaCl. Clarke and Lűpfert using fluorescence spectroscopy with fluorescent dyes [20], Tatulian using electrophoretic mobility method studied the effect of anions on the surface potential of phospholipid bilayers [8]. They have observed that the lipid membrane potential becomes more negative through adsorption of the anions in the order Cl^−^ < Br^−^ < NO_3_^−^ < I^−^ which is consistent with our surface charge density data. Nevertheless, to our knowledge, no published study in the literature has considered this adsorption as a function of pH.

**Figure 2:**
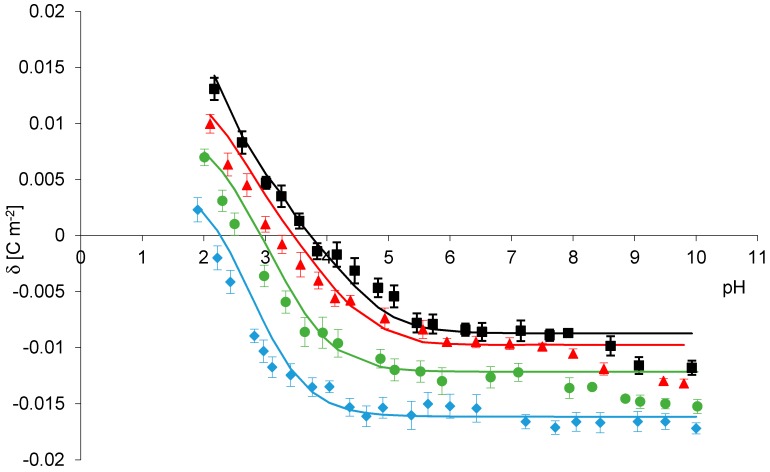
Surface charge density of phosphatidylcholine membrane versus pH of electrolyte solution. Points denote the experimental values, continuous line links the theoretical values NaCl (black), NaBr (red), NaNO_3_ (green), NaI (blue).

Comparison between the experimental and theoretical results for phosphatidylcholine liposomes in four different salts solutions NaCl, NaBr, NaNO_3_ NaI is given in Figure 2, respectively. Theoretical surface charge density values were determined based on four-equilibrium model (Theory section), by applying Equation (12) to the experimental data. Points denote experimental values, and continuous lines represent theoretical ones. In Figure 2 it is shown that the experimental points are in good agreement with theoretical lines in pH between 2 and 8 but diverge at pH > 8. The existence of such a difference may be due to the failure to take account experimental points, obtained at pH > 8 in the theoretical model. While conducting research, we always evaluate a scatter in the received data and at pH greater than eight measured values were characterized by a certain scatter. That is one reason we did not include them in our theoretical calculations.

Parameters characterizing the binding of ions to PC liposomal surfaces are summarized in Table 2.

The values of association constants K_AH_ K_ANa_, K_BOH_, K_BX_ for sodium chloride binding to phosphatidylcholine membranes were previously reported [22]. As can be seen from the table, the determined values of association constants of monovalent anions to trimethyloammonium group of phosphatidylcholine (K_BX_) range 0.076–0.36 [m^3^ mol^−1^]. These data indicate that the values considerably exceed those obtained by others [8,27]. Review of the literature in the topic allows noticing that binding constants of anions to phospholipids exhibit significant differences in the values not only between experimental methods, but also between experiments of various scientific groups using similar methods, which also noted Aroti [28]. The possible cause of the discrepancy between our data and literature has been explained in our previous work [10] may be the result of different defining the binding constant. We relate the concentration of solution ions to a unit of volume (mol m^−3^) while concentrations of phospholipid functional groups - to a unit of area (mol m^−2^).

Based on obtained both experimental (Figure 1) and theoretical data (Figure 2, Table 1), it can be concluded that effect of studied monovalent anions on the phosphatidylcholine membrane surface charge follows the Hofmeister series in the form Cl^−^ < Br^−^ < NO_3_^−^ < I^−^. Hence, among the anions, I^−^ shows the strongest interactions with phosphatidylcholine. Since the interaction between solution ions and functional groups of membrane lipids have an electrostatic character, the formed systems are ion pairs. Adsorption properties of sodium salts of anions belonging to the Hofmeister series to phosphatidylcholine liposomes increases with an increasing anion size. Chloride ions are small; the radius of Cl^−^ is equal 1.81 Å, whereas iodium ions are large; the radius of I^−^ is equal 2.16 Å, (Br^−^ = 1.95 Å, NO_3_^−^ = 1.89 Å) [29]. Larger anions adsorb to the membranes more than smaller ones due to their size, polarizability, and ion paring with choline group of the membrane [13]. Our findings are in accordance with data from the literature. Sachs et at., using molecular dynamic simulations, a method which in recent years provides useful information at the atomic level on local interactions of anions with lipid bilayers, found that anion penetration into zwitterionic lipid bilayers is mostly for large anions that can penetrate deeply into the bilayers [12,30].

Interactions between solution ions and lipids are important for the functioning of cells, as have influence on several physicochemical and electrical properties of the biological membrane, such as surface charge. Its value depends, among other things, on the composition of lipid membranes, the type and concentration of the electrolyte solution and pH. Obtained results confirm that changes in phosphatidylcholine membrane surface charge densities values are associated with the adsorption of both studied anions and sodium cation. Based on theoretical surface charge densities of PC membranes and the association constants of the PC functional groups with electrolyte ions confirm that the strongest adsorption is observed with I^−^ ions.

## 4. Materials and Methods

### 4.1. Materials

The L-α-Phosphatidylcholine (1,2-Diacyl-*sn*-glycero-3-phosphocholine, PC) from egg yolk Type XVI-E, ≥99% (TLC), lyophilized powder were purchased from Sigma-Aldrich (Poznań, Poland) and used without further purification. All sodium salts were of analytical grade quality (NaCl ≥ 0.99 mass fraction purity, NaBr ≥ 0.99, NaNO_3_ ≥ 0.99, NaI ≥ 0.99) from Sigma-Aldrich. The salts were prepared freshly before use. All solutions and cleaning procedures were performed with water purified by means of a Milli-Q plus water purification system (Millipore, MA, USA) with a resistivity of 18.2 MΩ cm. HPLC grade chloroform was also purchased from Sigma-Aldrich.

### 4.2. Preparation of Liposomes

Small unilamellar vesicles (SUVs) were prepared by the sonication method using an ultrasound generator UD 20 (Techpan, Puławy, Poland). Phosphatidylcholine was dissolved in chloroform (10 mg mL^−1^). The procedure was carried out in a glove box under an argon atmosphere to avoid the oxidation of the lipid. Then the organic solvent was evaporated under a gentle stream of argon to obtain dry lipid film. The lipid film was hydrated with appropriate electrolyte solution: NaCl, NaBr, NaNO_3_ NaI (155 mM). Liposomes were formed by sonicating the suspension using the ultrasound generator. Sonication was applied five times for 90 s. Since, during the process heat is liberated, cooling the suspension is necessary. It was carried out by using an ice bath (container with a mixture of ice and dry sodium chloride). The liposomes were freshly sonicated immediately before use.

### 4.3. Electrophoretic Mobility Measurements; Zeta Potential and Surface Charge Density Determination

Electrophoretic mobilities of liposome suspension were determined by means of a Zetasizer Nano ZS (Malvern Instruments, Malvern, UK), at 25 °C with the laser Doppler velocimetry technique. Disposable folded capillary cells (Malvern DTS 1070) were used to perform the experiment. Electrophoretic mobility measurements were carried out as a function of pH. The pH of the samples was conducted at room temperature, using a WTW InoLab pH 720 laboratory meter (WTW, Weinheim, Germany).

Liposomes suspended in four different sodium salt solutions (NaCl, NaBr, NaNO_3_ NaI) were titrated to the desired pH using acid or base. The pH was changed in range 2–9.5, every ±0.3 units. Six electrophoretic mobility measurements (each consisting of 100–200 runs with duration of 2 s), for every liposome sample, at a given pH value were performed. All experiments were conducted three times. Experimental data are reported as means ± SD from three independent measurements, statistical analysis were conducted using STAT30 program.

The zeta potential *ζ* of the liposomes were calculated, as in many papers [31,32] from the electrophoretic mobilities by application of Henry’s equation:

(15) $$\zeta =\frac{3\mu \eta }{2\varepsilon \varepsilon_{0}f\left(\kappa a\right)}$$

where: *μ* is the electrophoretic mobility *η* is the viscosity of the aqueous solution, *a* is the liposome radius, *κ*^−1^ is the Debye length, *ƒ*(*κa*) is Henry’s function. *ε*_0_ and *ε* are the permittivity of free space and the relative permittivity of the medium, respectively.

The experimental membranes surface charge densities were calculated from Equation (1) (Theory section), which is conversion of Smoluchowski equation for large non-conducting particles. If the particle size becomes large compared to the thickness of the double layer, then the particle and its double layer are treated as a parallel condenser [25]. We described the problem more in detail in the Theory (page 3).

### 4.4. Particle Size and Polysipersity Index Determination

The size and polydispersity index (PDI) of liposomes were determined using dynamic light scattering (DLS) method applied in Zetasizer Nano ZS equipment. Measurements were carried out at 25 °C. The size distribution of the particles was evaluated from intensity of the dispersed light, which is the basic parameter in the Zetasizer Nano ZS software.

## 5. Conclusions

The influence of electrolyte solution containing chaotropic anions on the physicochemical properties of model membranes is of great of biological significance. We have employed both experimental and theoretical approaches to describe binding of different anions from the Hofmeister series to a model membrane composed of phosphatidylcholine. Studies of biomimetic models are an invaluable source of knowledge about the processes occurring in natural membranes. Determined values, such as association constants may be used in the description of the interactions existing in membranes of living cells and their biophysical studies. Therefore, the significance of such studies may be specifically valuable for modern medical and chemical analysis and contribute to the progress in translating chemical theories into practice, providing an important and essential information understanding of mechanisms occurring in biological membranes.

## Figures and Tables

**Table 1:** Size, polydispersity, and zeta potential of phosphatidylcholine liposomes dispersed in different anionic media with concentration 155 mM.

Electrolyte	Liposome Size ± SD * (nm)	% Intensity	PDI	Zeta Potential (mV)
NaCl	215.5 ± 25.86	85	0.404	−5.73
	50.70 ± 5.46	15		
NaBr	198.7 ± 18.78	94.7	0.431	−6.33
	30.34 ± 2.23	5.3		
NaNO_3_	189.6 ± 19.85	93.9	0.532	−8.43
	30.04 ± 2.19	6.1		
NaI	163.0 ± 46.55	88.8	0.281	−10.27
	38.29 ± 6.62	11.2		

* SD: standard deviation.

**Table 2:** Association constants of phosphatidylcholine functional groups with monovalent ions (H^+^, Na^+^, OH^−^, Cl^−^, Br^−^, NO_3_^−^, I^−^).

Association Constants [m^3^ mol^−1^]				
Electrolyte	K_ANa_	K_AH_	K_BOH_	K_BX_
	(10^−1^ m^3^ mol^−1^)	(10^2^ m^3^ mol^−1^)	(10^9^ m^3^ mol^−1^)	(10^−1^ m^3^ mol^−1^)
NaCl [22]	2.30	7.17	3.35	0.76
NaBr	1.97	2.64	3.54	1.33
NaNO_3_	1.51	0.47	5.99	1.85
NaI	1.12	0.13	17.3	3.61
